# Polyester Resin–Quartz Composites in the Age of Artificial Intelligence and Digital Twins: Current Advances, Future Perspectives and an Application Example

**DOI:** 10.3390/polym18060753

**Published:** 2026-03-19

**Authors:** Marco Suess, Peter Kurzweil

**Affiliations:** 1 Gebrüder Dorfner GmbH & Co. KG, Scharhof 1, 92242 Hirschau, Germany; marco.suess@dorfner.com; 2 Department MBUT, University of Applied Sciences (OTH) Amberg-Weiden, Kaiser-Wilhelm-Ring 23, 92224 Amberg, Germany

**Keywords:** unsaturated polyester resin, quartz composites, digital twin, artificial intelligence, peak time evaluation

## Abstract

Unsaturated polyester resin (UPR)–quartz composites have become increasingly important in structural, sanitary, and architectural applications. However, their manufacturing processes still rely heavily on empirical knowledge. This review compiles recent developments in materials science, curing kinetics, and digital manufacturing, outlining a pathway toward data-driven, adaptive production of quartz-filled thermosets. The chemical and physical fundamentals of UPR polymerization are summarized, including the influence of initiator systems, filler characteristics, and thermal management on network formation. Challenges associated with highly filled formulations—such as viscosity control, dispersion, shrinkage, and exothermic peak prediction—are discussed in detail. Recent advances in digital twins (DTs) and artificial intelligence (AI) are reviewed, demonstrating how physics-based simulations, machine learning models, and hybrid mechanistic–data-driven approaches improve the prediction of rheology, curing behavior, and quality outcomes in thermoset polymer processes. A practical application example demonstrates the prediction of peak time in quartz–UPR composites using Random Forest and Gradient Boosting ensemble models. Two prediction scenarios are evaluated: Scenario A with gel time by Leave-One-Out cross-validation, and Scenario B without gel time, representing post-mixing and pre-process prediction contexts, respectively. Stratified bootstrap augmentation improves Gradient Boosting in both scenarios. Principal component analysis confirms that the curing process is governed by three independent physical dimensions: curing reactivity, thermal environment and resin thermal state.

## 1. Introduction

**Figure 1:**
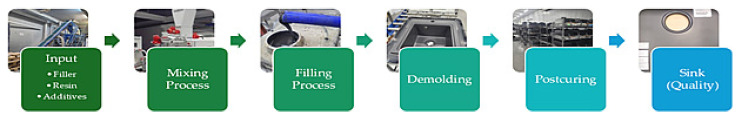
Steps for producing quartz-based kitchen sinks.

Polyester resin composites, particularly those based on *unsaturated polyester resins* (UPRs), have long played a critical role in the development of high-performance materials for structural and functional applications. Their manufacturing process still relies heavily on empirical know-how (Figure 1). Thanks to mechanical strength, chemical resistance, ease of processing, and cost-effectiveness, UPRs are the most widely used thermosetting polymer matrices in fiber-reinforced plastics, coatings, and adhesives [1,2].

A key factor in determining the final properties of UPR-based composites is the curing process, which primarily involves radical polymerization. The kinetics and mechanisms of this exothermic crosslinking reaction strongly influence the mechanical integrity, thermal stability, shrinkage behavior, and dimensional accuracy of the cured parts [3].

In recent years, filled UPR composites, particularly quartz-filled variants, have become increasingly relevant for applications requiring enhanced abrasion resistance, thermal durability, and surface hardness, such as in kitchen sinks, sanitary installations, and technical moldings [4,5]. However, the fabrication of these highly filled systems remains largely dependent on manual or semi-automated processes, resulting in variability in product quality, throughput, and resource efficiency.

Against this background, integrating *digital twins* (DT) and *artificial intelligence* (AI) into composite processing represents a paradigm shift. By combining real-time sensor data, physics-informed modeling, and machine learning algorithms, digital twin frameworks enable the predictive and adaptive control of the curing process, optimizing material properties and reducing defects under industrial conditions [6,7]. AI-driven analytics also support the identification of process anomalies, predictive maintenance, and quality assurance in complex manufacturing workflows [8].

Recent developments demonstrate a rapid convergence of artificial intelligence, *hybrid modeling approaches*, and industrial digital twin frameworks in composite manufacturing. Modern AI-driven digital twins combine physics-based simulations with machine learning models to enable continuous synchronization between the physical process and its virtual counterpart. This architecture enables real-time monitoring, predictive analytics, and autonomous process control across manufacturing systems, thereby supporting adaptive optimization and improving reliability in industrial production environments [9]. Additionally, emerging hybrid AI frameworks are integrating physics-informed neural networks, statistical learning methods, and process simulations to enhance predictive accuracy while reducing the need for experimental training data. These hybrid approaches facilitate the modeling of complex thermo-chemical curing processes and multi-scale material behavior in composite manufacturing systems, particularly when experimental datasets are limited or costly to obtain [10,11].

*Transfer learning and multi-fidelity learning framework* approaches derive knowledge from related material systems or simplified simulations for use in more complex industrial processes. These approaches significantly accelerate model training and improve predictive performance. This strategy is particularly valuable for curing simulations and process optimization in thermoset composites because generating extensive experimental datasets can be prohibitively expensive [12,13].

*Real-time diagnostic systems* are becoming increasingly important in composite manufacturing. Modern digital twins integrate sensor networks, computer vision, and machine learning algorithms to detect process anomalies, such as incomplete mold filling, porosity formation, or deviations in curing kinetics during production. These capabilities permit immediate corrective actions, improving product quality and reducing waste in industrial composite processing [14].

This article provides a comprehensive review of the current state of the art in UPR composite curing. It focuses particularly on *quartz-filled systems* and important developments in *thermoset materials* and processes that are like UPRs. It outlines the chemical and physical fundamentals of curing kinetics, strategies for tailoring polymerization behavior, and advanced computational models. The transformative role of digital technologies, particularly artificial intelligence and digital twins, in ushering in a new era of smart, reproducible, and efficient composite manufacturing is highlighted. It should be noted that it is impossible to include all relevant contributions in this review; therefore, emphasis has been placed on the most significant publications in this field to define the state of the art. Additionally, a brief conceptual framework is presented to demonstrate the potential of digital twin frameworks and artificial intelligence.

## 2. Materials and Applications of UPR Composites

Unsaturated polyester resins are among the most widely used thermosetting polymers in the composites industry. They are valued for their versatility, ease of processing, and favorable cost-performance ratio [1]. The *backbone materials* of UPRs are synthesized by the polycondensation reaction of diols, most commonly propylene glycol, neopentyl glycol or ethylene glycol, with a mixture of saturated and unsaturated dicarboxylic acids. The most common of these are phthalic anhydride, phthalic acid, and maleic anhydride [15].

For example:

$$n\;\mathrm{HOOC-CH=CH-COOH}\;+\;n\;\mathrm{HO-}CH_{2}CH_{2}\mathrm{-OH}\to \;\;\mathrm{HO}{\left[\mathrm{-CO-CH=CH-COO-C}H_{2}CH_{2}\mathrm{-O-}\right]}_{n}H+n\;H_{2}O$$

The resulting polyester prepolymer contains ester groups (-COO-) along its backbone and pendant unsaturated carbon–carbon double bonds. These C=C bonds act as reactive sites for *free-radical crosslinking*, which is typically initiated by organic peroxides such as methyl ethyl ketone peroxide and benzoyl peroxide (Bz-O-O-Bz), in the presence of a vinyl monomer such as styrene, which also functions as a reactive diluent [15,16]. For example:

$$(n-1)\;\mathrm{RCH=}CH_{2}\;+\;\mathrm{RCH=}CH_{2}\;+\;\mathrm{BzO}\cdot \;+\;H\cdot \;\to \;H{\left[\mathrm{CH}(R)\text{-}CH_{2}\text{-}\mathrm{CH}(R)\text{-}CH_{2}\right]}_{n}\mathrm{OBz}$$

The mechanism is shown in Section 3. The molecular architecture of UPRs offers a unique balance of rigidity, chemical adaptability and reactivity. Introducing *aromatic units*, such as those derived from phthalic acid, increases the rigidity of the molecular backbone and enhances resistance to chemical attack. The degree of *unsaturation* regulates the crosslinking density during curing, affecting mechanical strength, shrinkage behavior, and thermal resistance. Depending on the formulation and curing regime, UPRs typically achieve thermal stability up to approximately 150 °C [1,15]. They are also renowned for their excellent chemical resistance, particularly to water, dilute acids, and many organic solvents, and they exhibit strong adhesion to various substrates. This makes them ideal for use in coatings, adhesives, and laminates [3].

***Inorganic fillers.*** Despite these advantages, unmodified UPRs are brittle by nature and prone to scratches and microcracking when subjected to mechanical or thermal stress. Consequently, they are often reinforced with glass fibers or particulate fillers such as calcium carbonate (CaCO_3_) or quartz (SiO_2_), and sometimes blended with elastomeric tougheners to improve fracture toughness, reduce internal stress development, and enhance long-term durability [16,17,18].

To further tailor the performance of UPRs for structural or functional applications, inorganic fillers are widely employed. Among these, *quartz* (SiO_2_) is of particular interest due to its exceptional mechanical hardness, thermal stability, and chemical inertness. With a Mohs hardness of 7, quartz is highly resistant to abrasion and surface damage—a crucial attribute for applications exposed to repeated mechanical and chemical stress [17]. In both micronized and granular forms, quartz particles contribute significantly to the mechanical reinforcement of composites, acting as load transfer centers that increase compressive and flexural strength as well as chemical resistance. These improvements stem from the stress redistribution effect facilitated by the filler–polymer interface [17]. Moreover, quartz enhances thermal performance by increasing the heat deflection temperature and improving thermal conductivity—essential features for components operating at high temperatures or undergoing thermal cycling [17,19]. The addition of quartz also improves dimensional stability thanks to its low thermal expansion coefficient and stiffness, which reduces volumetric shrinkage during curing and service. In terms of surface properties, quartz contributes to enhanced abrasion resistance, which is particularly beneficial in consumer-facing applications such as kitchen and bathroom installations [4,17,18].

The properties of quartz-filled UPR composites depend strongly on the characteristics of the filler, including its particle size, morphology, and surface chemistry. While particles with a high aspect ratio can provide additional reinforcement, they may negatively affect flow behavior. Therefore, particle surface modification using silane coupling agents is frequently employed to improve the wetting properties and interfacial adhesion between the filler and polymer matrix. This leads to improved mechanical integration and reduced porosity [4,20,21,22,23,24,25,26].

Due to the performance advantages, quartz-filled UPR composites are increasingly used in a variety of commercial and industrial applications requiring robust mechanical properties, surface durability, and long service life. In kitchen sinks and countertops, these materials offer excellent scratch resistance, stain resistance, and visual appeal.

Their dense, non-porous surfaces are easy to clean and can be aesthetically tailored to resemble natural stone, making them competitive in the interior design market [23]. In sanitary engineering, quartz-filled UPRs are used for basins, shower trays and bathroom panels, where they are subjected to frequent contact with water, detergents and thermal fluctuations. The smooth, chemically resistant surface provided by the quartz filler promotes hygiene and longevity [4,24].

***Challenges***. In more technical fields, such as industrial moldings and machine components, these composites are used for their high compressive strength, dimensional stability and resistance to thermal and chemical degradation. Applications include chemical-resistant linings, pump housings, and panels for use in corrosive environments. Their performance, combined with their relatively low manufacturing cost, makes them a suitable alternative to ceramics and metals in many contexts [25]. Although quartz-filled UPR composites offer significant performance advantages, the processing of highly filled systems, particularly at filler contents exceeding 50–75 wt.%, introduces substantial challenges affecting manufacturability, product consistency and quality.

- One of the primary issues is *dispersion*. Quartz is much denser and more abrasive than UPRs and tends to agglomerate during mixing. If not properly stabilized, it can also quickly sediment over time. Inadequate dispersion can result in heterogeneities, local stress concentrations and reduced mechanical performance in the final product [23,26,27].
- Another key challenge is the increased *viscosity* of the resin–filler mixture. High viscosity complicates resin handling, mixing, and mold filling, especially in complex geometries. The use of reactive diluents or thixotropic additives can reduce viscosity and improve flow. However, these additives must be carefully selected to avoid adverse interactions with the curing process or degradation of mechanical properties [28].
- Air entrapment and *degassing* also become more difficult as viscosity rises. Bubbles introduced during mixing or filling may become trapped within the matrix or mold, leading to porosity, aesthetic defects, and weakened mechanical zones. Vacuum degassing, optimized mold design and proper treatment of molds with release agents are essential to mitigate this issue [29].
- Moreover, *cure inhibition* or delay may occur due to the adsorption of initiators onto the quartz surface or due to residual impurities on the filler surface interfering with radical propagation, or even a high filler content. To address these issues, precise control of initiator concentration, temperature, filler content and surface treatment (e.g., silane coupling agents) is necessary to ensure full conversion and a uniform cross-linked network [16,26,30]. The curing of quartz-filled UPR composites represents a highly complex, multivariate process governed by intricate interdependencies between chemical kinetics, filler characteristics, and processing conditions.

Despite this inherent complexity, current industrial practice still relies heavily on empirical knowledge and operator expertise. This highlights the urgent need for systematic approaches to unravel the underlying mechanisms and enable process optimization for greater reproducibility, efficiency, and material performance.

## 3. Polymerization of Unsaturated Polyester Resins

The curing of UPRs involves an exothermic, free-radical crosslinking polymerization that transforms the liquid polyester-styrene mixture into a rigid, three-dimensional thermoset polymer. The mechanism comprises: initiation, propagation and termination.

- The *initiation process* generates primary radicals by the redox decomposition of organic peroxides, such as methyl ethyl ketone peroxide [HOO-C(CH_3_)(C_2_H_5_)-O]_2_ (MEKP), in the presence of cobalt(II) octoate [H(CH_2_)_4_CH(C_2_H_5_)-COO]_2_Co (Equation (1)) [30,31].

RO–OH (MEKP) + Co^2+^ → RO**·** + OH^−^ + Co^3+^ + by-products ROOH + Co^3+^ → ROO⋅ + H^+^ + Co^2+^ (1)

- These peroxide radicals attack the C=C double bonds in both the unsaturated polyester and the styrene, creating reactive chain centers (Equation (2)) [30,31].

RO**·** + [–CO–CH=CH–CO-O(CH_2_)_2_O–]*_n_* → [–CO–ĊH–CH(OR)–CO-O(CH_2_)_2_O–]*_n_* (2)

- During *propagation*, the macroradicals incorporate additional unsaturated units to progressively form a cross-linked network (Equation (3)), e.g., by styrene (Ar=C_6_H_5_).

Ar–CH=CH_2_ + [–CO–Ċ=CH(OR)–CO-O(CH_2_)_2_O–]*_n_* → [–CO–CH(CH_2_ĊHAr)–CH(OR)–CO-O(CH_2_)_2_O–]*_n_* (3)

- *Termination* occurs via radical recombination (Equation (4)) or disproportionation (Equation (5)).

…–CH_2_–ĊH–R^1^ + …–CH_2_–ĊH–R^2^ → …–CH_2_–CH(R^1^)–CH(R^2^)–CH_2_–… (4)

…–CH_2_–ĊH–R^1^ + …–CH_2_–ĊH–R^2^ → …–CH=CH–R^1^ + …–CH_2_–CH_2_–R^2^ (5)

Overall, the decomposition of MEKP (Equation (1)) initiates polymerization, leading to gelation and network formation (Equation (6)). The exothermic reaction further accelerates conversion.

Initiation → Propagating radicals → Crosslinked polymer network(6)

The *curing* process exhibits strong auto-acceleration due to the Trommsdorff–Norrish (gel) effect: increasing viscosity hinders radical termination and promotes rapid crosslinking. Therefore, effective thermal management is crucial to prevent uncontrolled exotherms, hot spots, internal stresses, and structural defects [30,31,32,33].

## 4. Kinetics and Chemical Analysis

The curing kinetics and ultimate properties of UPRs are governed by several interrelated factors, with the type and concentration of the *radical initiator* being particularly decisive. Variations in initiator loading, typically reported in the range of 0.5–2.5 wt.% (5–25 g/kg) relative to the resin content, directly affect the onset temperature, gel time and crosslink density. While these ranges are well established in practice, the rationale behind their selection is often empirical, raising the question of whether these conventional loadings represent optimal compromises or merely workable ones. Temperature control during curing adds another layer of complexity: insufficient curing results in residual monomers and reduced mechanical integrity, whereas excessive thermal input risks degradation or volumetric shrinkage. In response, curing protocols often use staged temperature ramps and post-curing steps under elevated conditions to achieve full conversion and enhance thermal resistance. However, the extent to which such schedules systematically balance reactivity, internal stress development, and long-term performance remains unclear, indicating a need for more mechanistic, predictive approaches [34,35,36,37].

The incorporation of *fillers*, particularly at high loadings, introduces an additional level of complexity to UPR curing. On the one hand, fillers may act as thermal sinks, attenuating the exothermic reaction and potentially leading to spatially inhomogeneous cure fronts. On the other hand, insufficiently treated filler surfaces can restrict the mobility of reactive species or even inhibit radical propagation, making surface chemistry and dispersion critical determinants of the overall process. While surface modification strategies such as silane coupling are widely applied, the extent to which they consistently ensure interfacial compatibility and uniform network formation remains subject to debate.

To elucidate and model these interactions, a range of thermo-analytical and spectroscopic techniques has been employed; however, it remains questionable whether current methodologies capture the full multiscale dynamics of highly filled systems [26,37,38,39,40,41,42].

***Thermal analysis.*** Differential scanning calorimetry (DSC) is the most widely used technique for characterizing the curing behavior of UPR systems. It provides access to key parameters such as total reaction enthalpy, onset temperature, and reaction rate under both isothermal and dynamic conditions. From DSC data, kinetic models can be established to estimate activation energies and conversion rates across different degrees of cure. These models offer a valuable basis for understanding and comparing the influence of formulation, temperature control, and filler content. Moreover, when DSC is combined with complementary analytical methods such as thermogravimetric analysis (TGA) or Fourier-transform infrared spectroscopy (FTIR), a more detailed assessment of the underlying reaction mechanisms becomes possible, thereby supporting the development of more robust process and material models [22,34,35,36,38].

***Infrared spectroscopy*.** The degree of conversion in unsaturated polyester–styrene systems can be quantified by monitoring unsaturation depletion using Fourier Transform Infrared Spectroscopy (FTIR). Styrene C=C double bonds are identified by C–H out-of-plane bending vibrations at 912 and 992 cm^−1^, whereas polyester C=C groups are characterized by the absorption at 982 cm^−1^, which is assigned to *trans*-R-CH=CH-R structures. While styrene consumption can be determined directly from the reduction in the peak area at 912 cm^−1^, the quantification of polyester double-bond conversion is complicated by spectral overlap between the 982 and 992 cm^−1^ bands (Table 1).

Subtraction methods have therefore been applied to resolve these overlapping signals and to provide a more reliable estimation of polyester unsaturation conversion [38,39,40,41,42].

FTIR has further been employed to assess the terminal conversion of *methacrylate* end groups in vinyl ester (VE) resins diluted with styrene. Urban et al. [34] demonstrated that, under curing conditions at 26 °C, styrene undergoes homopolymerization to form atactic polystyrene segments that subsequently become covalently integrated into the polyester–styrene network. Dell’Erba et al. [35] investigated conversion profiles of styrene and polyester double bonds across the temperature range 336–363 K using FTIR, while Huang and Chen [36] reported on the influence of comonomer composition on the curing kinetics of unsaturated polyester resins, combining DSC with IR analysis. Complementary rheological studies have elucidated viscosity evolution and gel point determination, thereby providing critical insights into the relationship between chemical conversion, processability, and the definition of the processing window [38,39,40,41,42].

***Process control.*** Future research should focus on integrating spectroscopic conversion monitoring with rheokinetic modeling to achieve a more comprehensive description of curing behavior. Advanced approaches such as FTIR coupled with *multivariate statistical analysis* or *machine-learning*-assisted spectral deconvolution could overcome limitations related to peak overlap and manual subtraction. This would enable accurate real-time monitoring of unsaturation consumption. However, achieving consistent cure quality in manual or semi-automated manufacturing environments remains challenging due to variability in ambient conditions, handling, initiator dosing, and mixing efficiency. The inherent non-linearity and sensitivity of radical polymerization to thermal and kinetic disturbances further complicate process control in the absence of embedded monitoring systems. These challenges highlight the need for digitally enhanced control strategies such as sensor-based feedback loops and predictive modeling frameworks like *digital twins*, that integrate kinetic models with live process data to optimize cycle times, reduce defects, and translate laboratory insights into industrial robustness.

## 5. Quartz Composite Manufacturing

**Figure 2:**
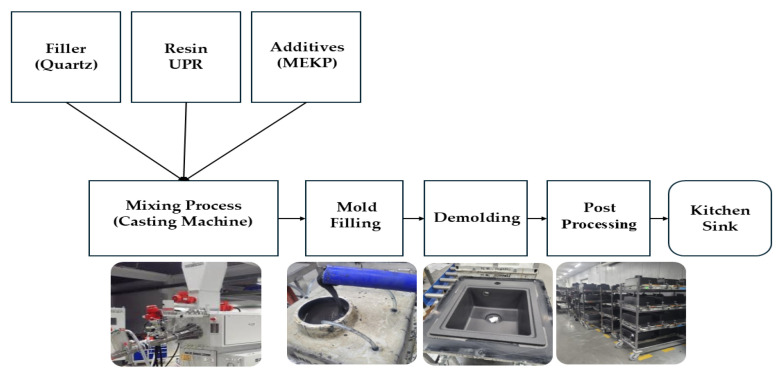
Materials and processes for quartz-composite manufacturing.

The manufacture of quartz composite sinks and solid surface materials, which are widely used in kitchen and sanitary installations, is a well-established industrial process. It involves blending UPR with pre-processed quartz sand and other minerals such as dolomite or silica, as well as curing agents, pigments, and auxiliary additives. This formulation is typically prepared in continuous casting systems (Figure 2) or blending pots fitted with suitable stirring equipment.

The result is a thixotropic yet flowable slurry with a very high filler content (typically exceeding 70 wt.-%). The slurry is then cast into glass fiber-reinforced molds [27,43,44,45].

During *mold filling*, the flow behavior of the quartz–UPR slurry is often enhanced via mechanical vibration because UPR exhibits shear-thinning, pseudoplastic rheological behavior [43]. The exothermic curing process within the mold leads to the formation of a dense, crosslinked thermoset matrix [3,4].

Critical challenges in this manufacturing process include maintaining the homogeneity of the filler dispersion, controlling the release of exothermic heat during polymerization, and minimizing the porosity of the final product by using vacuum curing processing and a proper time frame to release the material from the mold, as well as a final treatment of the thermoset matrix, known as post-curing.

These parameters are highly sensitive to both the formulation and processing conditions, necessitating stringent and precise process control throughout the production cycle [27].

## 6. Research Landscape in Thermoset Composites and Digital Manufacturing Technologies

This section provides a historical overview of thermosetting polymers, focusing not only on UPRs but also on thermosets in general, as well as casting processes, such as injection molding. Due to the scarcity of dedicated literature, a holistic perspective that includes related fields is adopted for an overview. Early developments in curing kinetics and process optimization are discussed to contextualize the evolution of the field. Additionally, modern advancements in digitalization, including digital twins and artificial intelligence, are integrated to demonstrate how data-driven approaches now complement classical experimental and modeling strategies to achieve a more thorough understanding of processes and materials.

### 6.1. UPRs and Their Composites

The study of unsaturated polyester resins (UPRs) has evolved significantly over the past four decades, driven by the need to optimize curing processes to enhance the mechanical performance and durability of composite materials.

***Cure kinetics***. One of the foundational works in this field was conducted by A. J. Rojas [46] in his 1981 publication ‘The curing of unsaturated polyester resins in adiabatic reactors and heated molds.’ Rojas was among the first to systematically analyze the thermal behavior of UPRs during curing, emphasizing the role of exothermic reactions in closed systems. His work established the foundation for understanding how the curing environment–specifically, the distinction between adiabatic and isothermal conditions–affects the rate and extent of cross-linking in polyester matrices [46]. The rate of cure is given by (Equation (7)):

(7) $$\frac{dx}{dt}=k\cdot c(I)\cdot {\left(1-x\right)}^{3}\exp\left(\frac{-16.6}{RT}\right)\;\;\;\;\;\;\;\;\;\;\;\;$$

*c*(I) is the initiator concentration (mol·L^−1^, e.g., MEKP), *x* is the dimensionless degree of cure, d*x*/d*t* is the conversion rate (s^−1^), (1 − *x*)^3^ reflects a third-order dependence of unreacted sites, *R* is the molar gas constant $8.314\;J\cdot mol^{-1}\cdot K^{-1}$, *T* is the absolute temperature (K), *k* is the empirical rate constant (L·mol^−1^·s^−1^). The exponential term describes the temperature dependence according to Arrhenius’ law because it was shown that the reaction rate increases linearly with initiator concentration, where cobalt octoate acts as an effective accelerator. This was the first approach to modeling the curing behavior of UPRs in a mold. It was also found that the adjustment of parameters allows targeted control of gel time, exothermic peak and curing behavior [46].

***NMR spectroscopy***. With experimental techniques advancing, a notable contribution came from K. Bergmann and K. Demmler [47] at BASF in Ludwigshafen, who employed nuclear magnetic resonance (NMR) spectroscopy to analyze the curing behavior of UPRs. Their research, titled ‘Investigation of the Curing Process of Unsaturated Polyester Resins Using NMR Measurements’, provided molecular-level insights into the degree of cure and the progression of cross-linking reactions, significantly enhancing the resolution of kinetic studies beyond calorimetric methods [47]. Bergman and Demmler distinguished between highly *mobile protons* (mainly from unpolymerized styrene), less mobile protons (from unreacted polyester chains), and *immobile protons*, which indicate a fully cross-linked, glass region in the polymer bulk. The importance of post-curing was also demonstrated by obtaining the difference in highly mobile protons before and after post-curing at elevated temperatures on a UPR with a glass transition temperature (*T*_g_) above room temperature. After curing at room temperature, 7–18% of the protons remained mobile; post-curing reduced this mobile fraction to 3–7%. Nevertheless, it was found that UPRs tend to heterogeneous domains in contrast to a uniform infinite cross-linked network [47].

***Process modeling***. Rai and Pitchumani [48] advanced the field in 1997 with their study ‘Optimal Cure Cycles for the Fabrication of Thermosetting Matrix Composites.’ Their work investigates how to design optimal cure cycles for thermosetting matrix composites with the goal of minimizing processing time while ensuring adequate material performance. The study combines a kinetic model of the curing reaction of thermosets, like epoxy and polyesters, with the heat transfer modeling across composite thickness. This allows the prediction of temperature and degree of cure profiles throughout the parts. The numerical simulations resulted in optimized cure cycles achieving the required degree of cure in a shorter time compared to conventional cycles without any loss of thermal and mechanical properties. Substantial *inhomogeneities in the cure degree* between surface layers and the core of thicker composite parts were observed. Due to the thermal lag and internal exothermic heat generation. The optimized cycles reduce these inhomogeneities by adjusting heating ramps and including dwell (hold) stages. It was demonstrated that part thickness, thermal conductivity, and reaction kinetics have a major influence on the shape of the optimal cure profile. Thicker specimens require slower heating or longer hold to avoid internal *temperature gradients*. The significance of this research lies in its structured methodology that couples cure kinetics with thermal analysis in real tools, enabling predictive modeling of the curing process for thermoset composites by numerical methods. This represented an important step toward simulation-driven process control of curing thermoset materials like epoxy and polyester resins [48,49].

***Response surface methodology***. In the 2010s, there was increasing attention to the curing behavior of unsaturated polyester resins (UPRs) in industrial applications, particularly in the manufacturing of *fiber-reinforced plastics* (FRPs). Waigaonkar et al. (2011) [16] conducted a study to investigate the practical implications of curing UPRs used in FRP components. The study focused on the effects of various process variables, including catalyst concentration (MEKP), accelerator concentration (Coct), glass fiber content, and calcium carbonate (CaCO_3_) load, on gel time and peak exothermic temperature during the curing process. The study revealed that increasing the *concentrations* of MEKP and Coct significantly reduces gel time, indicating a faster curing process. However, higher MEKP concentrations led to an increase in peak exothermic temperature, while higher Coct concentrations resulted in a decrease in peak temperature. Higher CaCO_3_ content increased the gel time and dampened the exothermic heat, suggesting that fillers absorb some of the reaction heat. Similarly, increasing *glass fiber content* also increased gel time and delayed the peak exothermic temperature, likely due to the heat absorption properties of the fibers. The study employed response surface methodology (RSM) to model the relationships between process variables and curing parameters. Regression equations were developed to predict gel time and peak exothermic temperature based on the selected variables. An optimization problem was formulated to achieve desired combinations of gel time and peak exothermic temperature within specified ranges, aiming for better product quality and productivity. Confirmatory experiments validated the predicted results, confirming the reliability of the developed models. This research provides valuable insights into the curing behavior of UPRs in FRP manufacturing. The developed regression models and optimization strategies offer a framework for controlling and predicting curing parameters depending on the recipe of the composite and enabling manufacturers to optimize process conditions for improved product quality and efficiency [16].

***Cure Progress Modeling***. Barakat et al. [50] examined the curing behavior of UPRs under varying processing conditions, using differential scanning calorimetry (DSC) to assess both isothermal and non-isothermal kinetics. The study applied an autocatalytic model to simulate cure progression, revealing that the cure rate decreased with temperature while the total heat of reaction increased. The term α is the *degree of cure*, dimensionless, ranging from 0 (uncured) to 1 (fully cured). Maximum degree of cure reached α = 71.25% ≈ 723 g/kg at 85 °C, whereas at 5 °C, limited thermal activation restricted crosslinking to about 12%. The findings underscore the importance of controlled processing conditions for achieving optimal curing and material performance in composite manufacturing. The proposed model provides a practical predictive tool. However, it is primarily based on idealized DSC measurements and may not fully capture real-world complexities such as thermal gradients, catalyst diffusion, or secondary reactions. Complementary characterization methods, including rheometry or NMR, could improve model accuracy and robustness for industrial applications [50].

***Post-Curing***. Silva et al. (2020) [51] investigated the influence of post-curing treatments on the static and viscoelastic properties of polyester resins commonly used in structural applications. The study employed a combination of curing protocols at room temperature, 40 °C, and 60 °C, with additional post-cure treatments at elevated temperatures over varying durations. Mechanical performance was assessed via three-point bending tests, while viscoelastic behavior, including creep and stress relaxation, was analyzed. Degree of cure and crosslinking were characterized using Fourier-transform infrared spectroscopy (FTIR) and differential scanning calorimetry (DSC). Results demonstrated that higher *curing temperatures* substantially enhance *mechanical properties*, with bending strength increasing by 36.5% at 40 °C and 88.6% at 60 °C, compared to room-temperature cured samples. Post-cured samples exhibited reduced creep and stress relaxation, indicating improved time-dependent performance. The study highlights the critical role of thermal post-treatment in achieving optimal crosslinking and mechanical stability [51].

***Kamal–Sourour Model***. Matůšková, Vinklárek, and Honzíček (2021) [52] developed a kinetic model to investigate the impact of various accelerators, specifically iron and vanadium compounds, on the room-temperature curing of UPRs. Their study aimed to identify non-toxic *alternatives to cobalt*-based accelerators commonly used in the composite industry. Gel time measurements were carried out to prove the catalytic efficiency of these metal-based acceleration systems. It was found that the type of accelerator strongly influences the curing kinetics of UPRs at room temperature. Some alternative based on iron or vanadium provides efficient acceleration while avoiding toxicity and discoloration of the Coct catalyst. The *concentration* of the accelerator directly affects the cure rate and the final degree of conversion. Higher concentration generally increased the reaction rate, and both iron- and vanadium-based catalysts can be effectively used in ambient cured systems, providing a safer and environmentally friendlier alternative to cobalt-based catalysts. The proposed kinetic model, which successfully described the curing behavior of UPRs under ambient conditions, is related to the kinetic model found by Kamal and Sourour [36].

(8) $$\frac{d\alpha }{dt}=k\left(T\right){\;\alpha }^{m}{\left(1-\alpha \right)}^{n}\;\;\;\;\;$$

Equation (8) describes a modified autocatalytic model, where the degree of conversion α represents the fraction of reacted double bonds, which is dimensionless and ranges from 0 to 1, and *t* denotes time (s). The rate of reaction is expressed as a function of a temperature-dependent rate constant, *k*(*T*) in s^−1^, which is often modeled using the Arrhenius equation, *k*(*T*) = *A*⋅e^−Ea/RT^, with *A* being the pre-exponential factor (s^−1^), *E*_a_ the activation energy (J·mol^−1^), *R* the molar gas constant (8.314 J·mol^−1^·K^−1^), and *T* is the absolute temperature (K). The exponents *m* and *n* (both dimensionless) represent the reaction orders for the autocatalytic and unreacted components, respectively. The term α^m^ reflects the autocatalytic behavior, indicating that the reaction accelerates as curing progresses due to the generation of reactive species such as free radicals. The term (1 − α)^n^ accounts for the declining number of unreacted double bonds, which slows the reaction as conversion increases. By fitting this model to experimental gelation and conversion data, the authors were able to predict how variations in accelerator type and concentration influence both the curing rate and the final degree of conversion at room temperature. While the study offers a promising approach to predict a cure model for further AI-based modeling and replacing toxic cobalt accelerators with eco-friendly materials, it primarily focuses on the initial gelation phase. It does not address long-term mechanical properties or durability of the cured UPRs [52].

***Shrinkage Control***. W. Li and L. J. Lee (2000) [53] investigated low-temperature curing strategies for UPRs by incorporating *thermoplastic additives*. Their study utilized dilatometry and morphological analyses to examine the effects of various additives on shrinkage control during curing. During polymerization, unfilled UPRs undergo a volume shrinkage of 7–10%. The incorporation of thermoplastic additives, such as *polyvinyl acetate* (PVAc), significantly reduced curing shrinkage to 4–5% by promoting phase separation and forming a co-continuous phase structure. This structural modification led to a reduction in the internal stresses and fissures typically associated with conventional UPR curing processes. These findings suggest that such additives can enhance the toughness and dimensional stability of UPRs without the need for elevated curing temperatures, offering a pathway toward energy-efficient manufacturing and shrinkage control [53].

***Sustainable Composites***. Pączkowski and Głogowska (2024) [23] explored the fabrication of hybrid composites by reinforcing UPRs derived from post-consumer PET recyclate with quartz particles. The study aimed to combine sustainability with enhanced material performance. Composite samples with varying quartz contents were produced and characterized for their mechanical properties, thermal stability, and microstructure. Incorporating *quartz fillers* led to substantial improvements in flexural strength, stiffness, and thermal resistance. Microscopic analysis showed uniform dispersion of the quartz within the polymer matrix, facilitating effective load transfer and reducing stress concentrations. The results indicate that recycled PET can serve as a viable polymer matrix for high-performance composites when combined with mineral reinforcements, offering a sustainable approach for engineering applications like quartz–polyester-composites [54].

The field of polyester resin-based composites has evolved significantly, integrating advanced curing strategies, reinforcement approaches, and predictive modeling, setting the stage for the adoption of artificial intelligence. Foundational studies on UPRs established the importance of thermal control, initiator concentration and curing kinetics, while modern techniques such as NMR, DSC, and rheometry have enabled real-time, molecular-level monitoring of crosslinking and post-curing behavior. Incorporation of fillers like quartz, recycled PET-based matrices or PVAc has demonstrated improved mechanical performance, thermal stability, shrinkage control and environmental sustainability, addressing both structural and ecological demands. Low-temperature curing strategies and non-toxic accelerators further enhance dimensional stability, toughness, and process safety. Together, these advances provide rich datasets suitable for AI-driven modeling, enabling predictive simulation of cure kinetics, shrinkage, and mechanical performance. Looking forward, coupling material characterization with AI tools offers the potential for real-time optimization of polyester–quartz composites, bridging laboratory insights with industrial-scale, energy-efficient, and sustainable manufacturing.

### 6.2. Digital Twins and Artificial Intelligence

In recent years, kinetic modeling and process optimization for thermoset systems or injection molding have been increasingly complemented by research on digital twins (DT) and artificial intelligence (AI) in composite manufacturing. Based on the established understanding of cure kinetics, thermal behavior and process–property relationships, current studies focus on leveraging simulation-driven digital replicas and data-driven learning algorithms to bridge experimental insights with predictive, adaptive manufacturing frameworks. This development reflects a paradigm shift from descriptive and semi-empirical models toward *integrated digital ecosystems*, where DTs provide real-time process monitoring and AI enhances optimization, enabling more efficient, reliable, and sustainable composite production.

***Digital Twins***. Fernández-León, Keramati, Baumela and González (2024) [55] presented a digital twin (DT) framework tailored for resin transfer molding (RTM), a widely used liquid molding technique for structural composites. The proposed DT specifically addresses the detection of *flow inhomogeneities*, most notably race-tracking channels, which enable resin to bypass fiber regions and cause dry spots and insufficient impregnation. Central to the framework are two deep learning–based surrogate models with encoder–decoder architectures: a disturbance detector that infers the permeability distribution of the textile preform in real time from pressure sensor data, and a predictive model that estimates key quantities of interest, such as resin flow front progression and internal pressure fields within the mold. Both models were trained entirely on synthetic data derived from high-fidelity multiphysics simulations based on Darcy’s law, thereby generating a wide range of representative scenarios. Once deployed, the surrogates achieved less than 1% error in pressure field predictions with response times below 50 milliseconds, enabling real-time performance. Experimental validation under varying race-tracking conditions confirmed the ability of the DT to reliably monitor and predict flow and pressure evolution. The study introduces two key innovations: the concept of an ‘instantiated digital twin’, which incorporates sensor data during manufacturing to dynamically mirror the actual process state, and the demonstration that models trained exclusively on simulation data can successfully operate in real-world experimental settings [55].

Sobhani et al. (2024) [56] introduced a hybrid modeling framework to improve the prediction of catalyzed *polymerization reactors*, where purely mechanistic models often fail due to nonlinearities, side reactions, and parameter uncertainties. Their approach integrates three components: a first-principles mechanistic model that captures known kinetics and mass balances, a multi-layer perceptron (MLP) *neural network* trained to learn the residual deviations between model and experimental data, and a *linear regression* module that accounts for systematic linear offsets. By combining physical interpretability with data-driven flexibility, the hybrid framework aims to enhance predictive accuracy across a broader range of operating conditions. The model was trained and validated on fed-batch polymerization reactor data, covering both main reagent concentrations and side product formation. Results show that, compared to traditional mechanistic modeling, the hybrid approach reduces mean absolute errors for reagent concentration predictions by approximately 84–86%. Furthermore, the maximum prediction error across all outputs remains within 3.5%, indicating reliable stability even under non-ideal experimental conditions. These findings highlight the capacity of the hybrid strategy to generalize beyond nominal reactor operation, effectively capturing disturbances, catalyst behavior, and nonlinear reaction dynamics that are difficult to model analytically. Hybrid predictive modeling seems to offer a robust and scalable pathway for process monitoring and control in polymerization systems, which can be transferred to thermosets in general. By combining mechanistic understanding with machine learning correction, the method delivers both accuracy and interpretability, providing a promising foundation for smart reactor operation and future integration with digital twin and AI-based manufacturing strategies [56,57].

Nasiri et al. (2024) [58] investigated a knowledge-based framework for developing digital twin models in smart *injection molding* processes. From a physical standpoint, injection molding exhibits strong similarities to classical thermoset resin processing and is closely related to it in terms of fundamental mechanisms. Traditional injection molding systems often operate in isolation, with each stage having its own monitoring and improvement initiatives. This lack of integration can lead to inefficiencies and quality issues. To address this, the authors propose a comprehensive DT approach that replicates the entire injection molding process, enabling real-time monitoring, fault detection, and predictive maintenance. The proposed DT model integrates various components, including fault detection systems, additive manufacturing, and system integration, to automate development activities. By leveraging knowledge engineering, data analysis, and data mapping, the DT model allows for proactive identification of potential issues and optimization of the manufacturing process. This approach not only enhances the efficiency and reliability of injection molding but also supports the transition towards Industry 4.0 standards by enabling smart automation and data-driven decision-making. The authors highlight the importance of establishing connections and communication among all stages of the injection molding process, as changes in one stage can impact others. By implementing DTs, manufacturers can achieve a more cohesive and responsive production system. However, the paper also acknowledges the challenges associated with implementing DTs, including the need for effective conception and integration with existing systems. Overcoming these challenges is crucial for realizing the full potential of DTs in molding processes [58].

Liu et al. (2025) [59] introduced a digital twin diagnostic framework based on cross-device transfer learning for industrial monitoring systems. Predictive models trained on one device or process configuration are adapted to other machines with minimal additional training data. This capability improves the scalability and deployment efficiency of digital twin systems across heterogeneous industrial environments. Transfer learning enables the DT model to maintain diagnostic accuracy even when applied to new equipment with different operating characteristics. Transferable DT architectures are particularly relevant in composite manufacturing environments, where similar curing or processing phenomena occur across different molds, reactors, or production lines. Cross-device transfer learning is a promising strategy for implementing scalable digital twin frameworks that can adapt to process monitoring and predictive diagnostics in thermoset composite production systems [59].

Silva et al. (2023) [60] introduce a framework for *real-time quality prediction* in thermoplastic injection molding aimed at zero-defect manufacturing. They integrated a smart shop floor architecture combining the ‘MES RAILES’ and the Zero-Defect Manufacturing Platform, so-called the ZDMP ecosystem, to harmonize data, deploy machine learning classifiers, and issue alerts before defects occur. MES RAILES is a modular and interoperable Manufacturing Execution System specifically developed for Industry 4.0 applications and deployed in the context of AI-based quality prediction. The pipeline includes data augmentation, Human in the Loop labeling, feature extraction, and supervised learning in the form of an artificial neural network with support vector machines. The system is applied across traditional and stretch blow injection processes and demonstrates increases in overall equipment effectiveness (up to 12%), reductions in downtime (up to 9%), and fewer nonconforming parts. Notably, the classifier can detect process deviations several cycles in advance, enabling corrective intervention. Limitations include the need for machine and product-specific model training and the data acquisition overhead. The design principles and system architecture established for injection molding are transferable to thermoset manufacturing, enabling similar integration of AI-based quality prediction within an Industry 4.0 framework [60].

***Machine Learning***. Rothenhäusler and Ruckdäschel (2023) [61] demonstrated how data-driven approaches can significantly accelerate the optimization of polymer thermomechanical properties. By integrating machine learning algorithms with experimental datasets, they established predictive models capable of efficiently estimating the *glass transition temperature* from formulation parameters such as resin composition, curing agent type, and stoichiometric ratios. Their methodology not only reduced the number of required experimental iterations but also provided insights into the underlying structure–property relationships governing thermal behavior. The study exemplifies how machine learning can be employed as a powerful tool in polymer design, enabling rapid formulation screening and guiding the development of sustainable resin systems with tailored performance characteristics [61].

Pai et al. [62] presented a systematic evaluation of contemporary machine learning methodologies and their implementation in polymer science. The authors delineated the applicability of diverse algorithms, ranging from linear and nonlinear regression models to advanced neural network architectures, for the prediction of physicochemical and mechanical properties across various polymer classes. Their synthesis of existing studies demonstrated that *deep learning* frameworks, particularly multilayer perceptrons and convolutional neural networks, exhibit superior capability in modeling the complex, multidimensional relationships between formulation variables, molecular structure, and macroscopic performance. Furthermore, the integration of ML-driven models with experimental and simulation data to enhance predictive robustness and generalizability was shown. The review highlighted the paradigm shift from empirical trial-and-error approaches toward data-informed design strategies, underscoring machine learning as a pivotal enabler for accelerating polymer development, optimizing processing conditions, and advancing the rational design of high-performance materials [62].

Julien Molina et al. [43] demonstrated the efficacy of *artificial neural networks* in capturing the complex rheological behavior of unsaturated polyester resin systems. Utilizing multilayer perceptron architectures, the authors established quantitative correlations between formulation parameters, such as monomer content, resin type, additive concentration and the resulting dynamic viscosity. The trained models exhibited high predictive accuracy and generalization capability, well performing conventional regression-based approaches. By systematically analyzing feature relevance and network performance, Molina et al. provided valuable insights into the nonlinear dependencies governing resin flow characteristics. Their findings underscore the potential of neural networks as robust predictive tools for replacing empirical viscosity measurements, thereby enabling more efficient formulation optimization and reducing experimental workload in industrial polymer development with a focus on thermosets [43].

Kun-Cheng Ke and Ming-Shyan Huang [63] investigated the application of multilayer perceptron neural networks for modeling and predicting product quality in injection-molding processes. By integrating process parameters such as temperature, pressure, and cooling time as network inputs, the authors developed predictive models capable of accurately forecasting key quality attributes, including dimensional accuracy and surface integrity. Their results demonstrated that MLP networks effectively capture the *nonlinear and multivariate relationships* inherent in polymer processing, outperforming traditional statistical methods. The study highlights the adaptability of neural network frameworks to complex polymer manufacturing scenarios and underscores their potential to optimize process conditions, reduce defect rates, and enhance overall production efficiency in both thermoplastics [63].

Focusing on filled thermosets, Nayak et al. [64] applied a hybrid approach in which the response surface methodology (RSM) was integrated with neural network computation to predict the erosion behavior of FRP composites in combination with waste marble dust. The RSM was employed to systematically design experiments and model the influence of formulation and processing parameters, while the neural network component was used to capture complex nonlinear relationships not addressed by the statistical model alone. This combination allowed for improved predictive accuracy and a more comprehensive analysis of parameter interactions affecting wear performance. The study emphasizes the effectiveness of coupling RSM with neural networks as a versatile framework for analyzing and optimizing the mechanical durability of mineral-filled polymer composites [64].

Zheng et al. (2026) [65] developed a hybrid PSO–FPA–BP neural network model to predict the friction and wear behavior of copper-free resin-based brake materials. Particle Swarm optimization (PSO) and the flower pollination algorithm (FPA) improve the training of a backpropagation (BP) neural network. This hybrid model captures complex nonlinear relationships between formulation parameters and tribological performance. While the study focuses on brake materials, this approach can be transferred to other thermoset composite manufacturing processes [65].

Fan Zhang et al. [66] employed an approach that integrated machine learning models with domain-specific expert knowledge to optimize the thermomechanical properties of polyester resins. Experimental datasets were combined with chemically informed descriptors to train predictive models capable of forecasting heat resistance and moldability, enabling the identification of optimal formulation and processing conditions. By leveraging both data-driven insights and prior material knowledge, the methodology enhanced predictive reliability and facilitated targeted improvements in resin performance. The study demonstrates the potential of *integrating machine learning with expert guidance* as an effective strategy for the rational design and optimization of high-performance polymer systems [66].

***Practical Consequences***. The referenced studies collectively demonstrate how combining mechanistic understanding with data-driven and digital twin-based approaches enables the derivation of reliable *predictive models* for composite systems, including quartz-filled thermosets. DT frameworks for RTM and injection molding illustrate that high-fidelity simulation data, real-time sensing, and deep-learning surrogates can be fused to detect process disturbances, infer material properties, and forecast flow or pressure evolution with high accuracy—capabilities directly transferable to modeling resin flow and impregnation behavior in quartz–composite manufacturing. Hybrid mechanistic–ML strategies show how kinetic and mass-balance models can be systematically corrected using neural networks to capture nonlinearities, catalyst effects, and deviations from ideal cure behavior, thereby improving the robustness of cure and process predictions for mineral-filled thermosets.

Further contributions stem from AI-driven quality-prediction systems and manufacturing-execution integrations, which highlight how *supervised learning* can anticipate defects and link process parameter variations to part-quality attributes—methodological principles essential for stabilizing processing of filler-rich quartz systems. Studies on ML-based property prediction demonstrate how neural networks and hybrid statistical–ML models capture complex structure-property and formulation-viscosity relations. These approaches can reduce experimental workload and enable targeted optimization of thermal, rheological, and mechanical behavior, all of which are key for deriving reliable material models for quartz composites.

The studies reviewed in this section demonstrate that, when combined with appropriate data preprocessing strategies, machine learning algorithms are the most promising approach for predicting curing behavior in thermoset composites. The next section applies these methods to a laboratory-scale evaluation of peak time prediction in highly filled quartz–UPR composites.

## 7. Application Example: Conceptual Framework

**Figure 3:**
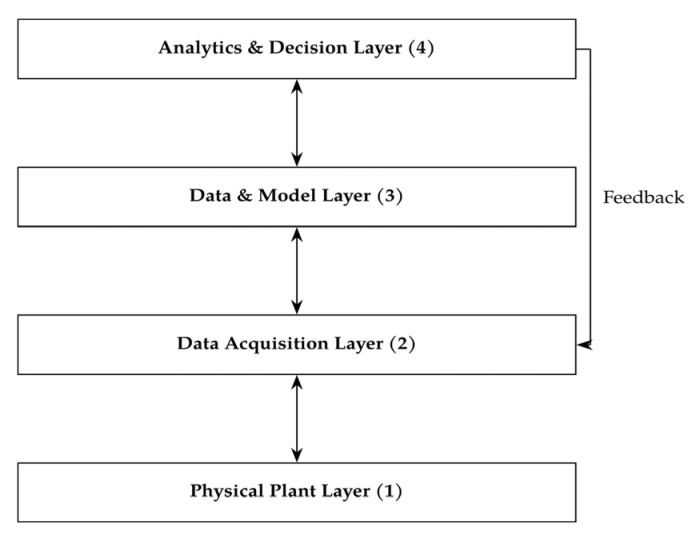
Digital twin framework.

The development of a *digital twin* to produce quartz composite sinks aims to represent, predict, and control the complex chemical–physics process involved in manufacturing unsaturated polyester resin composites. As described in Figure 3, a conceptual framework can describe such a process in theory before bringing it to life and establishing it in a real production.

In general, the digital twin consists of four main layers [37].

1. The *physical plant layer* encompasses the manufacturing hardware—continuous blending of ingredients and casting equipment, molds, and curing ovens. Comprehensive instrumentation is required to capture the system’s dynamic behavior, including flow meters, thermocouples or infrared sensors, in-line viscosimeters and vision systems for capturing recipes and visual appearance of the final product.
2. The *data acquisition layer* is responsible for real-time data collection, preprocessing, and control. Programmable logic controllers (PLCs) and edge gateways acquire high-frequency sensor signals, perform filtering and synchronization, and host localized DT proxies for low-latency control tasks like recipe feedback and feed rate stabilization.
3. The *data and model layer*, typically implemented on cloud or private computing infrastructure, manages the persistence storage and computational aspects of DT. It contains time series databases for real-time and historical data, model repositories encompassing both physics-based and machine learning models, and simulation engines for curing kinetics and rheology. This layer could also integrate surrogate models that enable near-real-time predictions based on high-fidelity offline simulations.
4. At the top, the *analytics and decision layer* hosts the real-time state estimator and optimization engines. The state estimator fuses incoming sensor data with physics-based model outputs through advanced data assimilation techniques to reconstruct latent variables such as local viscosity, temperature fields, and degree of cure. The optimization engine subsequently adjusts process parameters to balance competing objectives such as product quality, cycle time, and energy efficiency. DT’s supervisory control interface issues updated setpoints-e.g., recipe changes, mold temperature, or mixing speed to the physical plant, closing the control loop.

### 7.1. Use Case: Curing Kinetics and Peak Time Prediction

The prediction of time spans for exothermic reaction and demolding in quartz-unsaturated polyester resin (UPR) composites relies on the continuous advancement of kinetic and thermal modeling. Studies describing gelation and exothermic behavior as functions of initiator type, initiator and catalyst concentration, temperatures and filler content have provided a solid basis for understanding the curing kinetics of quartz-filled UPR systems. To achieve accurate kinetic modeling in highly filled UPRs, a comprehensive acquisition of process parameters is essential. This includes the temperatures of the filler, resin, and molds, ambient temperature, peroxide content, and overall matrix composition, as well as the temperature of the casting mixture prior to mold entry.

**Figure 4:**
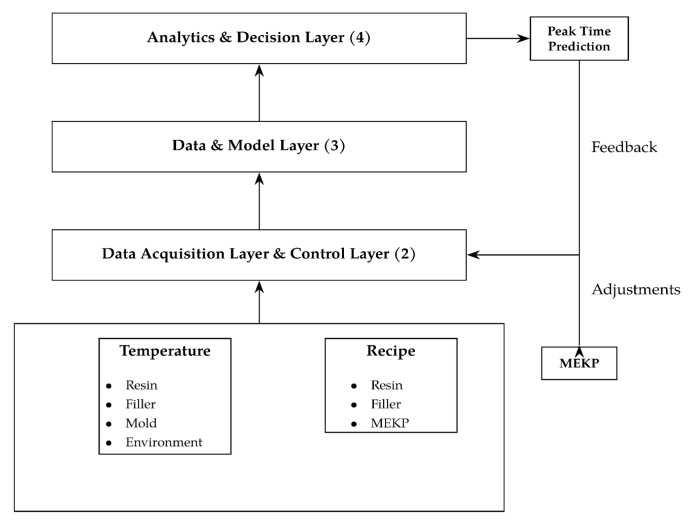
Digital twin framework for quartz–polyester composite production, focusing on peak time prediction and peroxide adjustment.

The objective of the setup in Figure 4 is to accurately predict the *exothermic peak time*, as significant volumetric shrinkage occurs immediately afterward. Delayed demolding during this phase can induce thermal stress, resulting in cracking and increased scrap rates. These effects can be mitigated using the implementation of a digital twin approach.

In the system described, thermal cameras and embedded sensors monitor the curing of cast kitchen sinks directly on the molds. Recipe parameters are logged by a CPU interface integrated into the casting machine. The acquired data is collected and synchronized within the physical plant and data acquisition layers (1 and 2) to ensure high-quality information streams. Subsequently, these data are transferred to the computational model layer 3, where *predictive models*, such as artificial neural networks, Random Forest or Gradient Boosting algorithms (see Section 7.2), analyze the kinetic behavior of the casting compound under real-time process conditions. The data model also incorporates geometric information, including mold and sink designs and wall thickness, which strongly influence peak temperature and curing time; thicker components tend to exhibit higher exothermic peaks [33].

Each mold is equipped with sensor tags that identify its current process stage. Once the exothermic peak is detected and a subsequent temperature decline is observed, layer 3 transmits a signal to layer 4, either triggering the mold to open or providing a visual signal to operators. Depending on the configuration of the model, the system can either predict peak times in advance or detect temperature decreases after the peak.

Unlike existing DT applications in RTM and injection molding, the proposed framework addresses the unique challenges posed by highly filled quartz–UPR composites. Conventional DT models cannot adequately capture strong local thermal gradients, nonlinear curing kinetics, and significant viscosity variations. However, this novel framework can handle geometric effects, high filler content, and coupled thermal–chemical–rheological phenomena. It allows for the accurate prediction of peak exotherm, adaptive demolding control, and real-time process optimization in a production environment.

### 7.2. Use Case: Laboratory-Scale Evaluation

The aim of this application example was to assess the feasibility of a digital twin framework under laboratory conditions by predicting the exothermic peak time (*t*_peak_) during the free-radical curing of highly filled quartz–unsaturated polyester resin (UPR) composites. Two ensemble-based machine learning algorithms—Random Forest (RF) and Gradient Boosting (GBR)—were trained and compared under two feature scenarios: Scenario A, which includes the gel time (*t*_gel_) as an input feature, and Scenario B, which excludes *t*_gel_ and thus represents a pre-process prediction setting that is more relevant for industrial real-time control.

#### 7.2.1. Experimental Dataset

A total of 76 curing experiments were conducted after a RANSAC-based outlier removal (see Section 7.2.2). RANSAC (random sample consensus) is a robust trial-and-error approach that removes noise and outliers from observational datasets and estimates parameters of a mathematical model [67,68]. For each trial, 300 g of material (resin and filler) was prepared according to the formulations specified in Table 2. Methyl ethyl ketone peroxide (MEKP) was added as the curing initiator, and the mixture was stirred at 1200 min^−1^ for one minute before being transferred into the mold.

The range of components and parameters was chosen to represent realistic conditions (Table 2). A small glass fiber-reinforced mold (120 mm × 1200 mm × 20 mm) was fabricated to simulate practical casting conditions.

**Figure 5:**
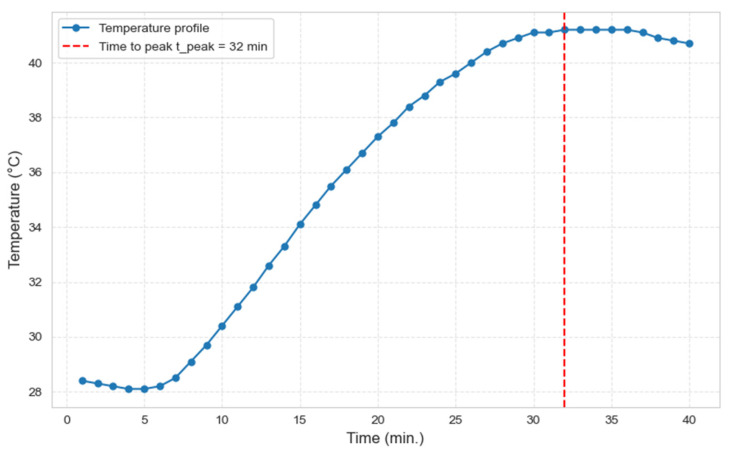
Temperature profile during the curing of a quartz–UPR composite in a mold.

An orthophthalic, pre-accelerated unsaturated polyester resin (Polipol^®^ 3506-X-A, Poliya, Istanbul, Turkey) was used as the matrix. Curing was initiated with MEKP (Peroxan^®^ ME 50 LX, Pergan, Bocholt, Germany), and Granucol^®^ Mix 10/2022 (quartz blend, particle size 0.1–0.8 mm) served as the filler material. Temperature profiles were recorded continuously using Type K thermocouples (Gel Instrumente AG, Oberutzwil, Switzerland) connected to a four-channel SD thermal logger (type 88598, Grainger, Lake Forest, IL, USA). The thermocouples were positioned within the resin to capture representative exothermic temperature profiles. The gel time was manually determined using a spatula. The peak temperature and corresponding peak time were extracted from the recorded temperature–time curves as shown in Figure 5.

#### 7.2.2. Numerical Evaluation

The recorded process variables served as input features for two ensemble-based regression models implemented in Python 3.12: a Random Forest (RF) model and a Gradient Boosting Regression (GBR) model. These algorithms were chosen for comparison of a *parallel* ensemble strategy (RF, in which independent trees vote by averaging) and a *sequential* ensemble strategy (GBR, in which each tree corrects the residual errors of its predecessors). Based on statistical analysis, three features were excluded from the input space: *filler content* was nearly constant at 70.0% across 66 of 76 trials, *resin content* was perfectly anti-correlated with filler content (r = −1.0), and 28 of 76 *filler temperature* values were imputed with the column mean due to missing measurements (Pearson *r* = 0.017 with *t*_peak_). Additionally, an RANSAC regressor with a residual threshold of 10 min was applied to identify and remove three statistical outliers (*t*_peak_ = 50, 61, and 84 min), reducing the dataset from 79 to 76 samples [67].

Two prediction scenarios were defined to investigate the influence of gel time on prediction accuracy (Table 3). Scenario A includes *t*_gel_ as an input feature (six features total) and represents a post-mixing prediction context. Scenario B excludes *t*_gel_ (five features total) and simulates a pre-process prediction that relies solely on controllable process parameters.

The *Random Forest algorithm* [54] constructs an ensemble of decision trees; each trained on a bootstrap sample of data with a random subset of features. The final prediction is obtained by averaging the individual tree outputs. This parallel construction reduces variance and provides robustness against overfitting, which is particularly advantageous for small datasets. *Gradient Boosting* [69] builds a predictive model by sequentially adding shallow decision trees, each of which is trained to minimize the residual error of the current ensemble. Through the iterative application of a learning rate of 0.05, the model gradually improves its fit while controlling for overfitting. Though each individual tree is a weak learner, the aggregated model captures complex nonlinear relationships in the data. All analyses were implemented in Python 3.12 using the libraries listed in Table 4, Table 5 and Table 6.

**Figure 6:**
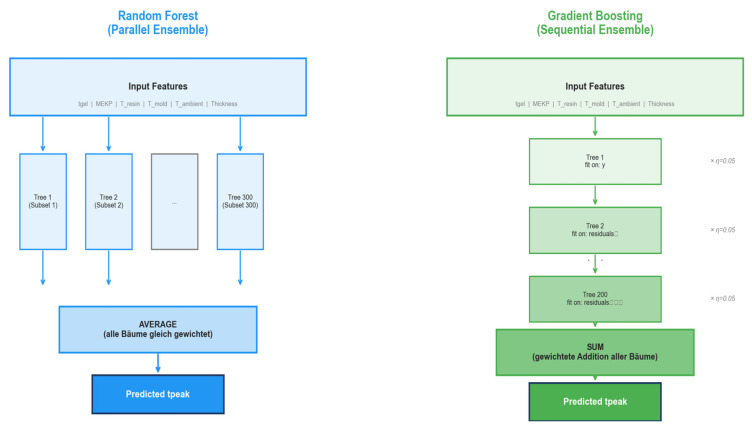
Model architecture: Random Forest (parallel ensemble, averaging) versus Gradient Boosting (sequential ensemble, weighted sum) for Scenario A. Scenario B uses the same architecture with five input features (excluding *t*_gel_).

Figure 6 illustrates the model architecture of the Random Forest and Gradient Boosting models. A hyperparameter tuning procedure was performed beforehand. The Random Forest builds 300 decision trees in parallel, with each trained on a bootstrap sample. The final prediction is the arithmetic mean of all the individual trees’ outputs. Gradient Boosting sequentially constructs 200 trees, with each successive tree fitting the residual error of its predecessor, scaled by the learning rate, η = 0.05.

The final prediction is the weighted sum *F*(x) = F_0_ + η·*h*_1_(x) + η·*h*_2_(*x*) + … + η·*h*_200_(x) [69]. Both architectures receive the same input features and produce the same output: the predicted peak time in minutes.

#### 7.2.3. Results of Random Forest and Gradient Boosting

**Figure 7:**
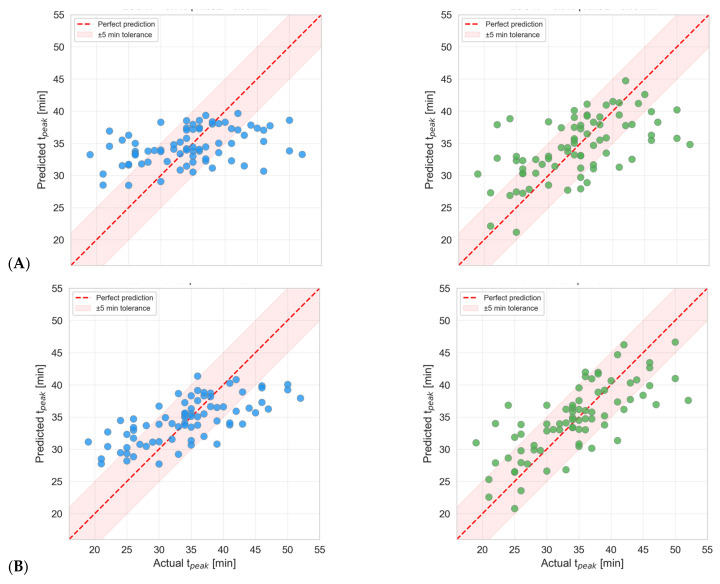
Regression parity plots: Left: Random Forests (blue), Right: Gradient Boosting (green). Scenario (**A**) (with gel time) and (**B**) (without gel time) from *n* = 76 experiments. Same abscissa.

Both models were evaluated using Leave-One-Out (LOO) cross-validation [70], in which each sample is predicted exactly once using a model trained on the remaining samples. This approach makes the most of the limited dataset and provides an unbiased estimate of generalization performance. Figure 7 presents the regression parity plots for all four model-scenario combinations. Each data point represents a single LOO prediction. The dashed red line indicates perfect prediction (*y* = *x*), and the shaded band denotes a ±5 min tolerance zone. In Scenario A, Gradient Boosting (R^2^ = 0.551, RMSE = 5.00 min) outperforms Random Forest (R^2^ = 0.430, RMSE = 5.63 min). In Scenario B, both models show reduced accuracy, with GBR (R^2^ = 0.340) again outperforming RF (R^2^ = 0.145). This confirms the *predictive importance of the gel time* feature.

**Figure 8:**
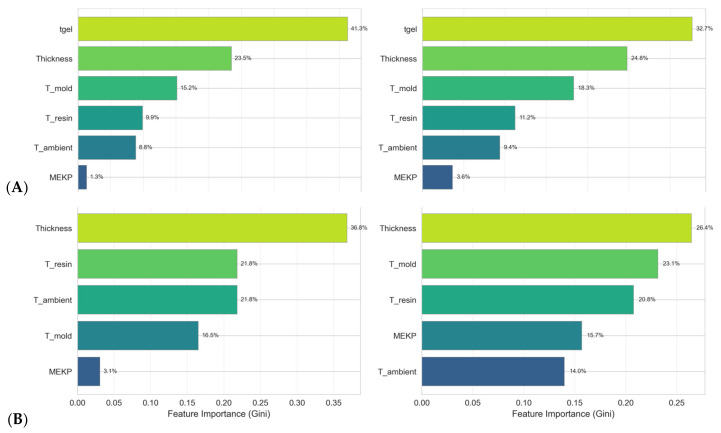
Feature importance (Gini-based) for Random Forest (**left**) and Gradient Boosting (**right**) for scenario (**A**) (with gel time) and (**B**). Original data, *n* = 76. Same abscissa.

Figure 8 shows the Gini-based feature importance [54] for both models. In Scenario A, *gel time* dominates in both RF and GBR models, with values of 41.3% and 32.7%, respectively, confirming its role as the strongest indicator of curing kinetics. Sample *thickness* ranks second (RF: 23.5%, GBR: 24.8%), reflecting the influence of thermal mass on heat dissipation. In Scenario B, thickness, resin and mold *temperature* become the primary predictors. *MEKP concentration* is relatively unimportant in Scenario A (RF: 1.3%), but becomes substantially more important in Scenario B (GBR: 14.0%), consistent with its direct role in radical initiation.

#### 7.2.4. Augmentation

Due to the limited number of samples (76) and the limited variation in MEKP concentration (only 1.5% was used in 68 of the original trials), the dataset’s effective information content is constrained. To address this issue, a stratified bootstrap augmentation strategy was employed. For each MEKP concentration group (0.8%, 1.5%, and 2.2%), samples were drawn with replacement and perturbed by Gaussian noise (σ = 5% of each feature’s standard deviation) to simulate the measurement uncertainty inherent to thermocouple-based temperature acquisition (±1.5 °C). This approach follows established practices in small-sample machine learning [71,72].

**Figure 9:**
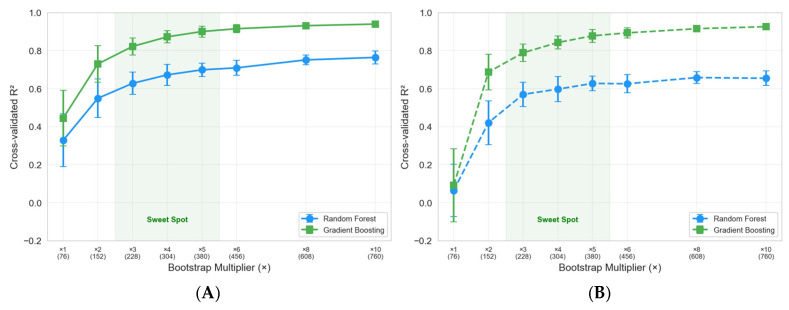
Augmentation sweep: cross-validated R^2^ (5 × 5 repeated CV) as a function of bootstrap multiplier for Random Forest and Gradient Boosting, Scenarios (**A**) ((**left**): with gel time) and (**B**) (**right**). ∎ Radom Forest, ∎ Gradient Boosting.

Figure 9 shows the augmentation sweep from ×1 (original) to ×10 for all four model-scenario combinations. Table 7 provides the corresponding cross-validated R^2^ values for the 5 × 5 repeated CV. The largest improvements occur between ×1 and ×3; thereafter, diminishing returns are observed. Gradient Boosting reaches R^2^ > 0.90 at ×5 for both scenarios, while Random Forest saturates around R^2^ ≈ 0.76 at ×10. A multiplier of ×8 (*n* = 608) was selected for subsequent analyses to balance improved statistical power against the proportion of synthetic data. Table 7 shows the effect of the stratified bootstrap multiplier on the cross-validated R^2^ for Random Forest and Gradient Boosting in Scenarios A and B. Increasing the multiplier from 1 (76 samples) to 10 (760 samples) steadily improves model performance, with Gradient Boosting consistently outperforming Random Forest. At an augmentation of ×8, Gradient Boosting achieves an R^2^ of 0.938 (root mean square error: 1.83 min) for Scenario A and an R^2^ of 0.922 (RMSE of 2.07 min) for Scenario B, while Random Forest reaches an R^2^ of 0.761 and 0.656, respectively. These results suggest that augmentation substantially improves predictive accuracy and reduces variability, with ×8–×10 being an effective level of augmentation for this small laboratory dataset.

**Figure 10:**
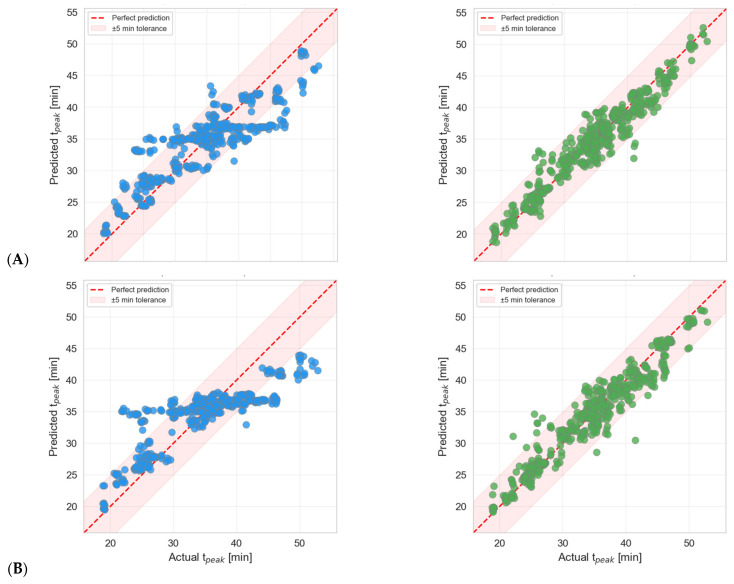
Regression parity plots for the augmented dataset (×8, *n* = 608) for Random Forest (● blue) (**left**) and Gradient Boosting (● green) (**right**) in Scenarios (**A**,**B**). Same abscissa.

**Figure 11:**
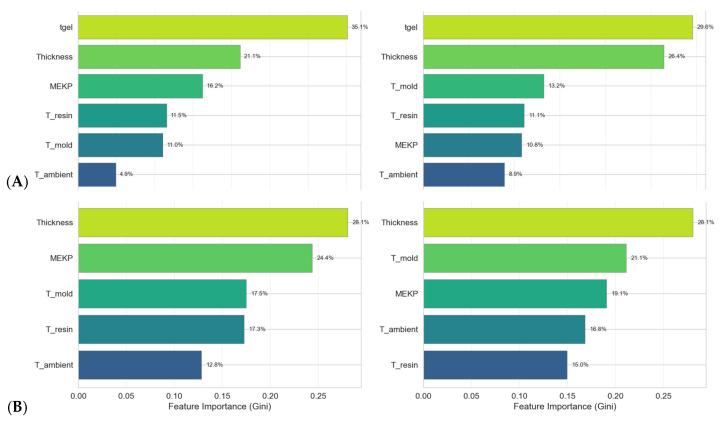
Feature importance (Gini-based) [48] for the augmented dataset (×8, *n* = 608). (**Left**): Random Forest Scenario. (**Right**): Gradient Boosting. Scenarios (**A**,**B**).

Figure 10 and Figure 11 and Table 8 illustrate the importance of the features for the models trained on the augmented dataset. Compared to the original dataset, there is a redistribution of feature contributions.

As shown in Table 9, MEKP’s importance increases substantially, rising from 1.3% to 16.2% for Random Forest Scenario A and from 14.0% to 19.1% for Gradient Boosting Scenario B. Conversely, gel time’s importance decreases slightly (RF A: from 41.3% to 35.1%), suggesting that the augmented data diminishes the dominance of individual features, offering the model a more balanced and representative feature space.

This redistribution suggests that augmentation not only increases the effective dataset size but also enables models to better capture the influence of underrepresented variables, thereby improving overall predictive performance and generalizability.

#### 7.2.5. PCA–Principal Component Analysis

**Figure 12:**
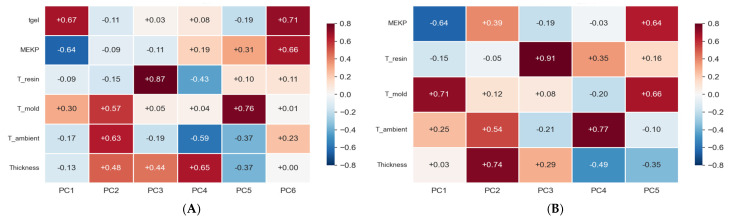
PCA loading heatmap for Scenario (**A**) ((**left**): with gel time) and Scenario (**B**) (**right**). The number of experiments is *n* = 76. Positive correlation in red (∎), negative correlation in blue (∎).

A principal component analysis (PCA) [73] was performed on the standardized feature matrices to assess the dimensionality and structure of the input space. PCA transforms the original, potentially correlated process variables into a set of linearly uncorrelated principal components (PCs), ordered by decreasing explained variance. This transformation (1) identifies the dominant directions of variation in the experimental data, and (2) evaluates whether reducing dimensionality is feasible without significant loss of predictive information. Figure 12 presents the PCA loading heatmap, which quantifies each original feature’s contribution to each principal component. Each cell in the matrix represents a loading coefficient (ranging from −1 to +1); the magnitude indicates the strength of the contribution; the sign indicates the direction to the respective PC.

In Scenario A, PC1 exhibits strong opposing loadings for gel time (+0.67) and MEKP concentration (−0.64), confirming that this component captures the *curing reactivity* axis. High gel time and low initiator concentration lie at one extreme, while rapid curing lies at the other. PC2 is dominated by the ambient temperature (+0.63) and the mold temperature (+0.57), representing the *thermal environment* of the casting process. Notably, the resin temperature dominates PC3 in both scenarios (loading = +0.87 in Scenario A, +0.91 in B), reflecting its unique thermal role in curing initiation. This orthogonality indicates that the resin temperature varies independently of the mold and ambient thermal conditions.

This is consistent with the practical observation that the resin is often stored and conditioned separately from the mold environment.

**Figure 13:**
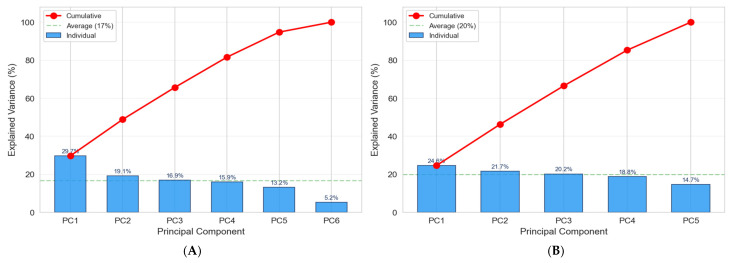
PCA screen plot showing individual (∎) and cumulative (●) explained variance for Scenario (**A**) ((**left**): with gel time) and Scenario (**B**) ((**right**): without gel time). Dashed line—**– –**: Average variance per component (Kaiser reference). *n* = 76 experiments.

Figure 13 shows the explained variance per component in the bar chart and the cumulative variance in the line graph. A distinctive feature of this dataset is the relatively even distribution of variance across all principal components: no single component exceeds 30%. This even distribution indicates that the *curing process* is genuinely multi-factorial, with no single dominant variable driving the system. In Scenario A, three PCs satisfy the Kaiser criterion (eigenvalue > 1.0) [64], together explaining 65.7% of total variance. In Scenario B, three PCs also exceed the threshold, explaining 66.5% of the total variance. The remaining PCs (PC4–PC6 in Scenario A, PC4–PC5 in Scenario B) individually account for 5–16% of the variance. While these components would typically be discarded under Kaiser-based dimensionality reduction, the correlation analysis presented below reveals that they carry substantial *predictive information* for the target variable *peak time*.

**Figure 14:**
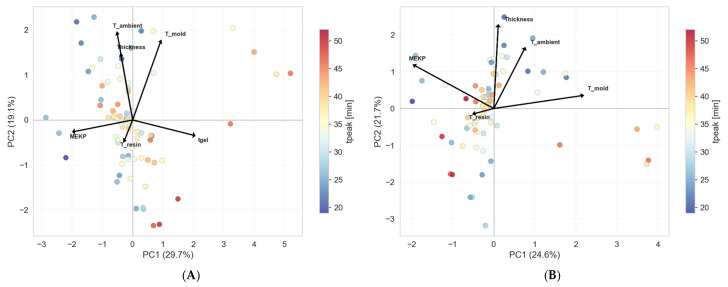
PCA biplot (colored by peak time) for Scenario (**A**) ((**left**): with gel time) and Scenario (**B**) ((**right**): without gel time). Arrows indicate feature loading vectors. *n* = 76.

Figure 14 presents the PCA biplots. Each data point represents a curing trial and is colored according to its measured peak time value. The loading vectors (arrows) indicate the direction and strength of each original feature in the principal component space. In Scenario A, PC1 separates the trials along the gel time–MEKP concentration axis (*curing reactivity*), and PC2 captures the *thermal environment* (mold and ambient temperature). The color gradient confirms that trials with high peak time values cluster in the direction of high gel time and low MEKP concentration. In Scenario B, removing the gel time fundamentally reshapes the PC space: PC1 is now dominated by the mold temperature (+0.71) versus MEKP concentration (−0.64), while sample thickness (+0.74) and ambient temperature (+0.54) define PC2. The color gradient in Scenario B is less clearly structured along the PC1–PC2 plane. This indicates that the strongest predictive variance for peak time is not captured by the first two components—a finding confirmed by the correlation analysis (Table 10).

***Scenario A*:** PC1, which captures the curing reactivity axis (gel time vs. MEKP concentration), exhibits the strongest positive correlation with peak time (*r* = +0.485). This is physically consistent: *longer gel times are associated with delayed peak exotherms*. PC5, which accounts for only 13.2% of the total variance, shows the second strongest correlation (*r* = −0.438). This component is dominated by the mold temperature (+0.76), suggesting a predictive influence on peak time that is partially masked in the original feature space. PC3 (resin temperature) shows virtually no correlation with peak time (+0.019). This indicates that, although resin temperature is an important process variable, its effect on peak time is largely mediated by other variables rather than acting as an independent predictor.

***Scenario B*:** Without gel time, the variance is distributed uniformly, and no single PC exceeds 25%. PC5, the smallest component with only 14.7% of the total variance, exhibits the strongest correlation with peak time (*r* = −0.578), substantially exceeding all other components. This component is dominated by the *mold temperature* (+0.66) and the MEKP concentration (+0.64). This indicates that the combined effect of mold temperature and initiator concentration (precisely the two controllable process parameters most relevant for industrial curing optimization) drives peak time variation in a direction that accounts for relatively little overall variance. Despite capturing the largest share of variance (24.6%), PC1 shows almost no correlation with peak time (+0.087). The decoupling of variance and predictive relevance has important implications for dimensionality reduction: applying a Kaiser-based PCA reduction (retaining only three PCs) would discard PC5 and thereby eliminate the strongest predictive signal.

***Results*:** The PCA results reveal that the curing process is governed by at least three independent physical dimensions: (1) *Curing reactivity*: Gel time and initiator concentration; (2) Thermal *environment*: Mold and ambient temperature; and (3) *Resin thermal state*. The strongest correlation with peak time was found in PC1 and PC5 in Scenario A, and in PC5 (r = −0.578) in Scenario B, indicating high- and low-variance principal components carry relevant predictive information about the peak time. This finding has direct consequences for machine learning model design: standard dimensionality reduction techniques based on variance thresholds (e.g., Kaiser criterion or 90% cumulative variance) would discard the most predictive directions in the feature space. Consequently, the Random Forest and Gradient Boosting models presented in Section 7.2.3 and Section 7.2.4 were trained on the full original feature sets rather than on PCA-reduced representations. Thus, the PCA serves primarily as an interpretive and diagnostic tool, confirming the physical structure of the input space, rather than as a preprocessing step for dimensionality reduction in this application.

#### 7.2.6. Algorithm Comparison

**Figure 15:**
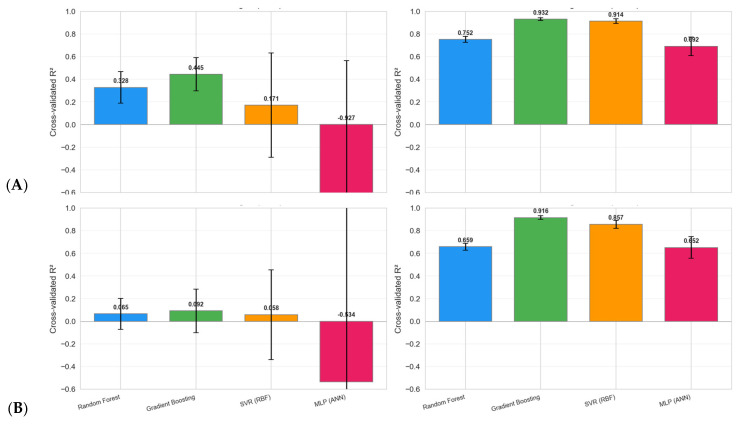
Comparison of four algorithms: cross-validated R^2^ on the original data ((**left**): *n* = 76) versus augmented data ((**right**): ×8, *n* = 608, solid) for Scenarios (**A**,**B**).

Figure 15 shows the CV R^2^ values of four algorithms on the same problem.

To contextualize the performance of RF and GBR, two additional algorithms were benchmarked: Support Vector Regression (SVR) [74] with a radial basis function kernel and a multilayer perceptron neural network (MLP) [75]. All four models were evaluated under identical cross-validation conditions (5×5 repeated CV) on both the original (*n* = 76) and augmented (×8, *n* = 608) datasets. The original data appear as faded bars alongside the augmented results (solid bars). The improvement Δ is annotated above each pair.

Table 11 summarizes the results. For the original data, GBR performs best in both scenarios (R^2^ = 0.445 and 0.092), followed by RF (0.328 and 0.065, respectively). SVR performs comparably to RF, while MLP fails entirely with negative R^2^ values. This confirms that *neural networks require substantially more training data* than was available here. With ×8 augmentation, all algorithms improve considerably. GBR achieves values of 0.932 and 0.916 for Scenario A and B, respectively, followed by SVR (0.914 and 0.857). MLP benefits most in absolute terms, improving from negative R^2^ values to 0.692 and 0.652 for Scenarios A and B.

#### 7.2.7. Key Findings

The laboratory-scale evaluation of peak time prediction in highly filled quartz–UPR composites yielded the following key findings:

- *Gradient Boosting* (GBR) consistently outperforms Random Forest (RF) across all scenarios and data conditions. On the augmented dataset (×8), GBR achieves RMSE prediction errors of about two minutes, which are within an industrially acceptable range for process monitoring.
- *Gel time* is the most important predictor in Scenario A; excluding it in Scenario B reduces regression depending on the model and data conditions. For example, LOO R^2^ drops from 0.551 to 0.340 for GBR using the original data and from 0.938 to 0.922 using the augmented data.
- *Stratified bootstrap augmentation* effectively addresses the limited sample sizes. The largest performance gains occur between ×1 and ×3, albeit diminishing improvements up to ×8 to ×10. A multiplier of ×8 provided Gradient Boosting with sufficient data to reach R^2^ > 0.90 in both scenarios while maintaining a conservative noise level (σ = 5% of standard deviation). This approach preserves the original feature distributions while simulating realistic measurement uncertainty.
- *Principal component analysis* (PCA) reveals that the curing process is governed by three independent physical dimensions: curing reactivity (gel time, catalyst concentration), the thermal environment (mold and ambient temperature), and the resin’s thermal state. Low-variance principal components (PC5) carry the strongest correlations with peak time, indicating that standard-based dimensionality reduction would discard critical predictive information.
- Among the four benchmarked algorithms, Gradient Boosting (GBR) offers the highest accuracy. On augmented data, it also exhibits the lowest cross-validation variance for Scenario A. A Random Forest (RF) exhibits the most stable performance on original data relative to its mean R^2^. SVR performs comparably to GBR on augmented data. MLP requires more data to achieve acceptable predictions.
- The complete data pipeline—from RANSAC-based outlier detection to stratified bootstrap augmentation, and further, to ensemble model comparison—provides a reproducible, statistically transparent framework for predicting peak times in highly filled thermoset composite systems. This framework supports developing digital twin strategies for quartz–UPR manufacturing processes.

## 8. Discussion and Further Perspectives

The progressive integration of DT and AI into the manufacturing of quartz–UPR composites marks a significant step toward realizing data-driven, adaptive, and sustainable production ecosystems. Traditional process control in thermoset composite manufacturing has been dominated by empirical knowledge and static curing models, which fail to adequately capture the nonlinear coupling between thermal, chemical, and rheological phenomena in highly filled resin systems. Digitalization enables a change in thinking—from descriptive and post-process monitoring to predictive and prescriptive control—where every stage of the casting and curing cycles can be optimized in real time.

*Digital twins* offer a virtual mirror of the physical production line, continuously updated with sensor and model data. By integrating multiple physical simulations of curing kinetics, exothermic heat evolution, and flow properties with live sensor feedback (temperature, viscosity, and infrared imaging), DT enables real-time prediction of peak exotherm and demolding time as seen in the conceptual framework. This capability allows manufacturers to prevent thermal stresses and reduce scrap through adaptive demolding strategies. Beyond process monitoring, DTs enable virtual experimentations, exploring the impact of formulation parameters, filler loading or catalyst concentration without interrupting production. In UPR–quartz systems, this digital replication is particularly relevant as their high filler content leads to strong local gradients in temperature and conversions that are difficult to capture experimentally.

AI augments the digital twin by extracting complex relationships from heterogeneous datasets, combining laboratory kinetics with in-line process measurements. Neural networks, MLPs, and hybrid physics-informed models have demonstrated superior capability in predicting curing behavior, viscosity evolution, and surface quality under varying environmental and recipe conditions. Implementing mechanistic or physics-based models, such as the Kamal–Sourour kinetic framework, could enhance predictive accuracy. These models provide a physically informed baseline that constrains machine learning models, reduces overfitting, and ensures consistency with known reaction kinetics. Incorporating these mechanistic insights enables the models to capture the underlying causal relationships in the curing process, thereby improving robustness and generalizability across different resin formulations and processing conditions.

- A laboratory-scale evaluation of peak time prediction in highly filled quartz–UPR composites provides preliminary evidence that supports the feasibility of DT–AI integration. *Gradient Boosting Regression* (GBR) outperformed Random Forest consistently across scenarios, achieving promising prediction accuracies for industrial relevance. Nevertheless, due to the limited size of the laboratory dataset, there are significant concerns regarding the applicability of these models to full-scale production. A limited sample size increases the risk of overfitting, and performance metrics may not be applicable under different environmental conditions, batch variations, or equipment configurations.
- *Gel time* was found to be the most influential predictor. While this highlights the importance of kinetic parameters for accurate prediction, it also reveals the model’s strong dependency on a variable that is usually unavailable in industrial processing. In practical manufacturing environments, determining gel time in real time is rarely performed, so predictive models must operate without direct access to this parameter.
- Although *stratified bootstrap augmentation* improved model robustness and reduced variance, synthetic augmentation cannot fully substitute for diverse experimental data. To improve model robustness, a broader experimental design is necessary that incorporates variations in MEKP concentration, filler loading, and process parameters. Additionally, the current laboratory dataset does not represent industrial effects such as equipment drift and strong thermal gradients, which could limit the model’s predictive reliability when transferred to full-scale production.
- *Principal component analysis* (PCA) identified three independent physical dimensions —curing reactivity, thermal environment, and resin thermal state—underscoring the importance of retaining low-variance components for accurate prediction. However, dimensionality reduction based on small datasets risks discarding subtle yet critical effects, and PCA insights may not be applicable to larger, more heterogeneous production datasets.
- *Neural network* approaches, including MLPs, required substantially more data to achieve acceptable performance. This emphasizes that AI-assisted models are strongly constrained by the availability of experimental data. Thus, despite promising lab-scale results, the robustness and scalability of AI-enhanced DT frameworks in real-world, large-scale thermoset production remain unverified.

The complete pipeline—from RANSAC-based outlier detection, to stratified bootstrap augmentation, to ensemble model comparison—provides a reproducible framework for laboratory-scale DT development. However, translating it to industrial settings requires careful consideration of sensor reliability, environmental noise, batch-to-batch variability, and scale effects, as these factors could significantly impact predictive accuracy and operational robustness. Future implementations of DT-AI architecture will enable closed-loop manufacturing, where process adjustments are made automatically based on predictive feedback. However, the current findings suggest that transitioning from lab-scale validation to full-scale deployment is challenging and requires extensive testing under real manufacturing conditions. Sensor-driven monitoring of exotherm and rheology provides information for AI-driven control decisions that adjust the concentration of peroxide or the cure ramp profile in real time. However, sensor degradation, limited spatial resolution, and extreme thermal gradients in high-filler systems remain major challenges.

Beyond process control, DTs and AI can foster sustainability through intelligent material design. By linking compositional parameters (e.g., recycled PET-based UPR matrices, bio-based monomers, eco-friendly accelerators) with predicted thermal and mechanical performance, AI models enable data-driven formulation optimization. Digital twins can simulate life cycle impacts and energy consumption associated with different process routes, promoting environmentally responsible manufacturing. Nevertheless, quantitative sustainability predictions depend on precise process models and validated life cycle assessment (LCA) data, both of which are limited for highly filled quartz–urethane prepolymer (UPR) composites.

Recent studies increasingly emphasize the role of artificial intelligence and digital twin technologies in enabling sustainable manufacturing strategies within the polymer and composite industries. However, these studies are often based on simulations or small-scale experiments, and there is a lack of large-scale empirical validation to confirm predicted CO_2_ savings and resource efficiencies in industrial environments.

***Outlook.*** While the potential of AI- and DT-enabled manufacturing is widely recognized, several key challenges remain. Acquiring reliable data in harsh thermoset processing environments, especially in highly abrasive quartz–polyester systems, requires robust sensor technologies, durable embedding strategies, and systematic calibration procedures. However, hybrid modeling frameworks that integrate first-principles curing kinetics with data-driven learning approaches lack methodological standardization, limiting their scalability. Future research efforts should therefore prioritize unsaturated polyester–based composite systems to evaluate robustness and reproducibility of DT-AI approaches at industrial scales.

The convergence of DTs and AI triggers a new era for quartz–UPR composite manufacturing. By merging the rigor of mechanistic modeling with the adaptability of data-driven prediction, these technologies pave the way for smart, autonomous, and sustainable composite production. Nevertheless, current laboratory evidence suggests that these technologies are still in the early stages of validation. Their true impact on large-scale manufacturing, sustainability, and process reliability has yet to be critically assessed.

## 9. Conclusions

The evolution of quartz–unsaturated polyester resin composites from empirically optimized materials toward digitally engineered products represents a decisive turning point in composite manufacturing. Traditional process control strategies, which are largely based on static models and operator expertise, are being supplemented and partially replaced by dynamic, data-driven frameworks. These frameworks can link formulation chemistry, process kinetics, and product performance in real time.

This review has demonstrated that the integration of digital twin (DT) technologies and artificial intelligence (AI) provides a powerful foundation for predictive and adaptive control of highly filled thermoset systems. The combination of multi-sensor data acquisition, mechanistic kinetic models, and machine learning algorithms enables the virtual replication of manufacturing processes, thereby allowing the prediction of critical events such as peak temperature, flow properties, and demolding. Coupling these digital representations with real-time feedback allows manufacturers to reduce scrap rates, improve reproducibility, and optimize energy consumption. This supports smart and sustainable production strategies.

Integrating laboratory-scale experimental data with AI frameworks is an initial step toward the intelligent, adaptive, and sustainable manufacturing of quartz–UPR composites. Using a dataset of 76 RANSAC-cleaned curing trials, experimental validation demonstrates that ensemble-based models can predict peak time with high accuracy (R^2^ = 0.938 for Gradient Boosting on augmented data) and that gel time, sample thickness, and thermal boundary conditions, such as mold temperature, are the dominant predictive features. Principal component analysis confirms the existence of three independent physical dimensions that govern the curing process. However, the limited dataset and laboratory-scale conditions necessitate the careful interpretation of these results. Further research, together with larger and more diverse datasets, is necessary to fully validate and quantify the potential of AI-driven and digital twin–based approaches for sustainable composite manufacturing.

Moreover, the implementation of AI-driven models extends beyond process optimization. Data-centric approaches facilitate the exploration of new formulations incorporating recycled or bio-based raw materials, non-toxic accelerators, and alternative fillers, thereby promoting a circular and environmentally responsible composite industry. The development of next-generation UPR composites with tailored mechanical, thermal, and aesthetic properties may be further accelerated by machine learning-supported materials design and life-cycle simulation.

Despite these advances, several challenges remain. The development of standardized digital infrastructures, robust sensor networks, and interpretable hybrid models is crucial for transferring laboratory-scale concepts into industrial applications. Ensuring data interoperability, cybersecurity, and model transparency will also be vital for achieving widespread adoption within Industry 4.0 and emerging Industry 5.0 frameworks.

Looking ahead, the fusion of digital twins (DTs), artificial intelligence (AI), and advanced materials science has the potential to enable self-optimizing, resilient, and sustainable manufacturing ecosystems for quartz–urethane prepolymer (UPR) composites. These smart systems will replicate and control physical processes, continuously learning and evolving to bridge the gap between materials research and intelligent production. This integration could transform quartz–UPR composites from conventionally engineered materials into adaptive, data-informed products of the digital age.

## Figures and Tables

**Table 1:** Specific FTIR absorption spectra for UPRs [38,39,40,41,42].

C=C in:	Wavenumber (cm^−1^)	Wavenumber (cm^−1^)
Styrene	912	992
Polyester	–	982

**Table 2:** Summary of the formulations and experimental variations, with peroxide (MEKP) amounts calculated relative to the resin mass.

Component/Parameter	Range	Unit
Filler Granucol^®^ 10/2022	68–75	% = 10 g/kg
Resin Polipol^®^ 3506-X-A	25–32	% = 10 g/kg
MEKP Peroxan ME 50 LX	0.8/1.5/2.2	% (relative to resin)
Temperature of resin	14.3–35.0	°C
Temperature of mold	13.0–47.0	°C
Temperature of filler	18.3–26.5	°C
Time to gelation (*t*_gel_)	1.0–16.0	min
Ambient Temperature	15.3–27.0	°C
Thickness of material	8.4–14.4	mm

**Table 3:** Feature sets of the two prediction scenarios.

Scenario	Features	*n*
A (with *t*_gel_)	*t*_gel_, MEKP, *T*_resin_, *T*_mold_, *T*_ambient_, thickness	6
B (without *t*_gel_)	MEKP, *T*_resin_, *T*_mold_, *T*_ambient_, thickness	5

**Table 4:** Software environment.

Library	Description
pandas	Data manipulation
NumPy	Numerical Operations
scikit-learn	Machine learning models
matplotlib	Visualization
seaborn	Visualization

**Table 5:** Adjusted hyperparameters used for the Random Forest and Gradient Boosting models.

Parameter and Data Type	Random Forest	Gradient Boosting
X numpy.array (n_samples × n_features)	*t*_gel_, MEKP, *T*_resin_, *T*_mold_, *T*_ambient_, thickness	*t*_gel_, MEKP, *T*_resin_, *T*_mold_, *T*_ambient_, thickness
Y numpy.array (n_samples)	Target variable: PeakTime	Target variable: PeakTime
n_estimators	300	200
max_depth	4	3
min_samples_leaf	5	5
max_features	0.7	–
random_state	42	42

**Table 6:** Model output parameters.

Output Parameters	Type	Description
y_pred	numpy.array	Predicted peak time for test data [min]
y_train_pred	numpy.array	Predicted peak time for training data (overfitting assessment)
R^2^	float	Coefficient of determination (cross-validated)
R^2^_train	float	Training R^2^ (used to calculate overfitting gap)
RMSE	float	Root mean squared error (min)
MAE	float	Mean absolute error (min)
feature_importances_	numpy.array	Gini-based relative importance of each input feature
oob_score_	float	Out-of-bag R^2^ estimate (RF only)
staged_predict()	generator	Prediction after each boosting stage, 1→200 (GBR only)
train_score_	numpy.array	Training loss per boosting iteration (GBR only)
LOO predictions	numpy.array	Leave-One-Out predicted values for all *n* samples
CV R^2^ (mean ± std)	float	Mean and error from repeated 5 × 5 cross-validation
Residuals	numpy.array	Actual versus predicted values (min)

**Table 7:** Effect of stratified bootstrap multiplier on cross-validated R^2^ (±standard deviation).

Multiplier		Random Forest		Gradient Boosting	
	*n*	A	B	A	B
×1	76	0.328 ± 0.139	0.065 ± 0.137	0.445 ± 0.146	0.092 ± 0.192
×2	152	0.549 ± 0.102	0.421 ± 0.115	0.730 ± 0.097	0.687 ± 0.095
×3	228	0.628 ± 0.059	0.570 ± 0.064	0.823 ± 0.045	0.789 ± 0.047
×4	304	0.673 ± 0.055	0.598 ± 0.065	0.873 ± 0.032	0.843 ± 0.034
×5	380	0.699 ± 0.036	0.628 ± 0.038	0.901 ± 0.029	0.878 ± 0.034
×6	456	0.710 ± 0.040	0.626 ± 0.048	0.916 ± 0.021	0.894 ± 0.026
×8	608	0.752 ± 0.026	0.659 ± 0.031	0.932 ± 0.013	0.916 ± 0.016
×10	760	0.765 ± 0.034	0.655 ± 0.038	0.940 ± 0.010	0.926 ± 0.012

**Table 8:** Prediction of the peak time using Leave-One-Out cross-validation on original and eightfold augmented data.

	Original Data		Augmented Data	
Model	*R* ^2^	RMSE	*R* ^2^	RMSE
Random Forest Scenario A	0.430	5.63 min	0.761	3.61 min
Random Forest Scenario B	0.145	6.90 min	0.656	4.33 min
Gradient Boosting Scenario A	0.551	5.00 min	0.938	1.83 min
Gradient Boosting Scenario B	0.340	6.06 min	0.922	2.07 min

**Table 9:** Feature importance (%) for both models and scenarios on the original data (*n* = 76) and eightfold augmented data (*n* = 608).

	Original Data				Augmented Data			
Feature	Radom Forest		Gradient Boosting		Random Forest		Gradient Boosting	
Scenario	A	B	A	B	A	B	A	B
*t*_gel_	41.3	–	32.7	–	35.1	–	29.6	–
Thickness	23.5	36.8	24.8	26.4	21.1	28.1	26.4	28.1
*T*_resin_	9.9	21.8	11.2	20.8	11.5	17.3	11.1	15.0
*T*_ambient_	8.8	21.8	9.4	14.0	4.9	12.8	8.9	16.8
*T*_mold_	15.2	16.5	18.3	23.1	11.0	17.5	13.2	21.1
MEKP	1.3	3.1	3.6	14.0	16.2	24.4	10.8	19.1

**Table 10:** PCA results for Scenario A (with gel time; 6 features; *n* = 76 experiments) and Scenario B.

Scenario	PC	Eigenvalue	Variance %	Cumulative %	Dominant Feature	Correlation with Peak Time
A	PC1	1.806	29.7	29.7	Gel time (+0.67)	+0.485
	PC2	1.162	19.1	48.8	Ambient temperature (+0.63)	−0.222
	PC3	1.026	16.9	65.7	Resin temperature (+0.87)	+0.019
	PC4	0.969	15.9	81.6	Thickness (+0.65)	+0.322
	PC5	0.801	13.2	94.8	Mold temperature (+0.76)	−0.438
	PC6	0.317	5.2	100.0	Gel time (+0.71)	−0.005
B	PC1	1.247	24.6	24.6	Mold time (+0.71)	+0.087
	PC2	1.098	21.7	46.3	Thickness (+0.74)	−0.227
	PC3	1.024	20.2	66.5	Resin temperature (+0.91)	+0.014
	PC4	0.953	18.8	85.3	Ambient temperature (+0.77)	+0.331
	PC5	0.745	14.7	100.0	Mold temperature (+0.66)	−0.578

**Table 11:** Summary of algorithm comparison: CV R^2^ on original and augmented data.

Algorithm	Original A	Augmented ×8 A	Original B	Augmented ×8 B
Random Forest	0.328	0.752	0.065	0.659
Gradient Boosting	0.445	0.932	0.092	0.916
SVR (RBF)	0.171	0.914	0.058	0.857
MLP (ANN)	−0.927	0.692	−0.534	0.652

## Data Availability

The original contributions presented in this study are included in the article. Further inquiries can be directed to the corresponding author(s).

## Abbreviations

The following abbreviations are used in this manuscript:

AI	Artificial Intelligence
ANNs	Artificial Neural Networks
BPO	Benzoyl peroxide (Bz-O-O-Bz)
CaCO_3_	Calcium Carbonate
Coct	Cobalt Octoate
CV	Cross Validation
DT	Digital Twin
DSC	Differential Scanning Calorimetry
FTIR	Fourier Transform Infrared Spectroscopy
FRPs	Fiber-Reinforced Plastics
GBR	Gradient Boosting Regression
IoT	Internet of Things
LCA	Life Cycle Assessment
LOO	Leave-One-Out Cross Validation
MEA	Mean Absolute Error
MEKP	Methyl Ethyl Ketone Peroxide
MES	Manufacturing Execution System
MLP	Multilayer Perceptron
MSE	Mean Squared Error
NMR	Nuclear Magnetic Resonance
PC	Principal Component
PCA	Principal Component Analysis
PET	Polyethylene Terephthalate
PVAc	Polyvinyl Acetate
QPCs	Quartz-Reinforced Polyester Resin Composites
R^2^	Coefficient of Determination
RANSAC	Random Sample Consensus
RF	Random Forest
RMSE	Root Mean Squared Error
RTM	Resin Transfer Molding
SVR	Support Vector Regression
OOB	Out-of-Bag (estimate)
UPR	Unsaturated Polyester Resin
VE	Vinyl Ester
ZDMP	Zero-Defect Manufacturing Platform
